# Impact of prescription size on statin adherence and cholesterol levels

**DOI:** 10.1186/1472-6963-7-175

**Published:** 2007-10-25

**Authors:** Holly A Batal, Mori J Krantz, Rita A Dale, Phillip S Mehler, John F Steiner

**Affiliations:** 1 General Internal Medicine, Denver Health and Hospital Authority, 777 Bannock St., Box 0148, Denver, CO 80204, USA; 2 Cardiology, Denver Health and Hospital Authority and Colorado Prevention Center, 777 Bannock St., Box 0148, Denver, CO 80204, USA; 3 University of Colorado Health Sciences Center and Colorado Health Outcomes Program, 4200 E 9th Avenue, Denver, CO 80220, USA; 4 Colorado Prevention Center, 789 Sherman Street, Suite 200, Denver, CO 80203, USA

## Abstract

**Background:**

Therapy with 3-Hydroxy-3-methylglutaryl Co-enzyme A reductase inhibitors (statins) improve outcomes in a broad spectrum of patients with hyperlipidemia. However, effective therapy requires ongoing medication adherence; restrictive pharmacy policies may represent a barrier to successful adherence, particularly among vulnerable patients. In this study we sought to assess the relationship between the quantity of statin dispensed by the pharmacy with patient adherence and total cholesterol.

**Methods:**

We analyzed a cohort of 3,386 patients receiving more than one fill of statin medications through an integrated, inner-city health care system between January 1, 2000 and December 31, 2002. Our measure of adherence was days of drug acquisition divided by days in the study for each patient, with adequate adherence defined as ≥ 80%. Log-binomial regression was used to determine the relative risk of various factors, including prescription size, on adherence. We also assessed the relationship between adherence and total cholesterol using multiple linear regression.

**Results:**

After controlling for age, gender, race, co-payment, comorbidities, and insurance status, patients who obtained a majority of fills as 60-day supply compared with 30-day supply were more likely to be adherent to their statin medications (RR 1.41, 95% CI 1.28–1.55, *P *< 0.01). We found that statin non-adherence less than 80% was predictive of higher total serum cholesterol by 17.23 ± 1.64 mg/dL (0.45 ± 0.04 mmol/L).

**Conclusion:**

In a healthcare system serving predominantly indigent patients, the provision of a greater quantity of statin medication at each prescription fill contributes to improved adherence and greater drug effectiveness.

## Background

Adherence to medications such as statins is a critical component in the treatment of chronic illness. Adherence can be defined as the extent to which patients follow the instructions that they are given for prescribed treatments [1]. Although many patient and provider factors promote successful adherence, these are often difficult to modify. By contrast, barriers to adherence in the delivery system itself are more easily addressed [2].

Pharmacy policy is a critical step in modulating medication adherence behavior, especially in health care systems that provide incentives to fill medications within the system. In such systems, pharmacy records provide a means to ascertain the pattern and timing of drug acquisition, as well as providing information on related factors, such as co-payment and prescription fill size. Refill adherence is associated with improvements in health outcomes among patients with hypertension and coronary heart disease [3,4]. Conversely, approximately 10% of all hospital admissions are attributable to a patient's inability to follow advice regarding their drug therapy plan [5]. Pharmacy policies, such as increasing the amount of co-payment or reducing the size of prescription pills, are designed to reduce pharmacy costs by increasing patient cost-sharing or reducing waste. However, these strategies may not lower overall costs if accompanied by a decrease in drug adherence [6,7]. Few studies have directly evaluated the effect of prescription size on adherence [8]; intuitively it seems that an increase in the amount of medication dispensed could positively influence patients' ongoing medication adherence and reduce total pharmacy costs by reducing dispensing services.

Because system issues such as pharmacy and reimbursement policies can impact adherence behavior, we sought to determine the effect of prescription size on patients' adherence to hyperlipidemia therapy. As a drug class 3-Hydroxy-3-methylglutaryl Co-enzyme A reductase inhibitors (statins) are ideally suited for the assessment of adherence. They are taken once daily, administered indefinitely, and are associated with a reduction in CHD events in both primary and secondary prevention settings [9]. The effectiveness of statins is easily gauged by measuring cholesterol levels. To determine whether a 60-day statin dispensing policy also influenced clinical outcomes, we also assessed the effect of prescription size on cholesterol levels in patients undergoing treatment for hyperlipidemia. We hypothesized that a larger dispensed prescription size would be associated with greater drug adherence and decreased total cholesterol levels, even after controlling for baseline clinical and sociodemographic characteristics.

## Methods

This retrospective cohort study examined patients receiving care and medications at Denver Health Medical Center (DHMC) over a three-year period. DHMC is a public safety net hospital serving a predominantly minority and indigent population, which provides nearly one third of the uncompensated medical care for the state of Colorado. Denver Health (DH) is an integrated health care system including community health clinics, subspecialty care clinics, and an acute care hospital (DHMC). Outpatient clinics and inpatient services are served by a single integrated pharmacy system. DH actively screens patients for enrollment in an indigent care prescription program, which provides discounted drug benefits to eligible patients. Although this is not an insurance benefit, it does allow patients to obtain their medications for free or a small co-payment. In addition, the pharmacy participates in many medication assistance programs that provide medications to qualifying persons free of charge.

Patients were eligible for study inclusion if they had received at least two prescriptions for any statin between January 1, 2000 and December 31, 2002. Each patient's study period was defined from the day of his or her first statin medication fill until the day of his or her last statin prescription fill. Thus, the number of days in the study for each patient varied, and our investigation was not limited to patients newly initiated on statin therapy. Study patients' records were linked to laboratory data through each patient's unique medical record number. The Colorado Multiple Institutional Review Board approved this study.

### Measurements

#### Adherence

Our indirect measure of adherence was pharmacy refills, given that patients seen in Denver Health had a substantial financial incentive to fill their medications within the system. In such closed pharmacy systems, pharmacy records provide a virtually complete measure of medications obtained [10,11]. Each patient's adherence score was calculated as their days of drug acquired divided by their days in the study (days from first prescription fill to last prescription fill), using the methodology of Steiner et al [10]. High adherence was defined by an adherence score of greater or equal to 80% and non-adherent as an adherence score of less than 80%. This cut point is conventional in the adherence literature [12-14] and yields measures of association that are easily interpreted. Adequate treatment of hyperlipidemia does not require perfect medication adherence, making the 80% cut off an appropriate maker for adherence.

#### Achievement of target cholesterol levels

Serum cholesterol data was obtained from Denver Health laboratory records. The cholesterol level analyzed was the last available cholesterol value obtained during or not more than 6 months after the patient's study period.

### Independent variables

#### Days supply

The main independent variable of interest was days supply of statin received at each prescription fill. The amount of statin medication per fill dispensed at our institution during the study period was usually either a 30- or 60-day supply of medication. At the time of this study, a provider could write for up to a 60 day supply of medication and the patient would be charged a single co-payment for that fill. Patients were grouped into either the 30- or 60-day category based on whether the modal size of their prescriptions was less than 45 (30 day) or greater than or equal to 45 (60 day).

### Sociodemographic and clinical variables

Age was defined as the patient's age at the midpoint of the study period. Insurance status was defined as the method most frequently utilized by the patient. Co-payment was measured for each prescription fill. The co-payment required for each patient and each prescription varied with a patient's financial status and insurance status. Patients were considered to have no co-pay during the study if the median co-pay for all of their statin prescriptions was equal to $0. Gender and self-defined race/ethnicity were also collected from pharmacy records. Overall burden of chronic disease was controlled for by totaling the number of comorbidities identified in the last 6 months from the patient's pharmacy records, using modified Chronic Disease Score methodology [15].

### Statistical analysis

All analyses were performed using SAS statistical software version 8.2 or higher (SAS Institute, Inc., Cary, NC). Two-sided *P *values of less than or equal to 0.05 were considered statistically significant. Demographic variables were compared between adherence groups using the chi-square statistic for categorical variables and a two-sample *t *test for continuous variables. Continuous variables are presented as means ± standard deviation (SD) and categorical variables are presented as number (n) and percent per group.

Log-binomial regression was utilized to determine the relationship between days supply and adherence while controlling for other factors. Additional independent variables assessed in the log-binomial regression model included gender, age, race/ethnicity, insurance status, co-payment, and number of co morbidities. Because the reporting of odds ratios for common outcomes may overestimate the magnitude of association, we used log-binomial regression to directly calculate adjusted risk ratios [16].

We assessed whether receiving a 30- or 60-day supply more frequently had an effect on the patient's final serum cholesterol level using a two-sample t-test. Multiple linear regression was used to identify factors impacting the patient's final cholesterol value. We modeled the data so that the intercept predicted the cholesterol value for a 50 year old white male, insured with no co-payment, who received mostly a 60-day supply of statin medication, and who was adherent. We then ran a set of simplified the models to calculate the indirect effect of statin adherence on cholesterol level to explore whether days supply was a strong mediator in the process.

A paired analysis comparing adherence was performed on the subgroup of patients that received at least 2 consecutive fills each of 30- and 60-day statin medication. An adherence score was calculated for each of the longest consecutive strings of 30- and 60-day supplies for each patient and the scores were compared using a paired *t *test.

## Results

We obtained data for 5,073 patients who had filled any statin prescription during the study period. After excluding those who had filled only one statin prescription, 3,386 patients were available for analysis, encompassing over 27,000 statin prescription fills. Total cholesterol values were available for 3,292 of these patients. Dichotomizing adherence at greater than or equal to 80%, 1,610 of the 3,386 (47.5%) patients were classified as adherent with statin therapy. Adherent and non-adherent patients are compared in Table 1. In univariate analysis neither insurance status nor the obligation of a co-payment showed a significant relationship to adherence. A higher percent of patients in the adherent group received 60-day statin fills compared to the non-adherent group (81.2% vs. 70.2%, *P *< 0.001). Moreover, final serum cholesterol levels were lower in the adherent patients (mean (std) = 176.8 (42.2) vs. 195.9 (50.8), *P *< 0.001).

**Table 1:** Relationship between Patient Characteristics and Statin Adherence

	**Total (n = 3386)**	**≥ 80% adherence (n = 1610)**	**< 80% adherence (n = 1776)**	***P *Value**
**Age (years), mean (SD)**	57.8 (10.9)	58.7 (10.7)	57.0 (11.0)	<0.001
**Gender, n (%)**				
Female	1932 (57.1)	881 (54.7)	1051 (59.2)	0.009
Male	1454 (42.9)	729 (45.3)	725 (40.8)	
**Race, n (%)**				
Hispanic	1584 (46.8)	683 (42.4)	901 (50.7)	
White	919 (27.1)	515 (32.0)	404 (22.8)	<0.001
Black	638 (18.8)	276 (17.1)	362 (20.4)	
Other	245 (7.2)	136 (8.5)	109 (6.1)	
**Insurance, n (%)**				
Insured	989 (29.2)	485 (30.1)	504 (28.4)	0.26
Uninsured	2397 (70.8)	1125 (69.9)	1272 (71.6)	
**No. of comorbidities, mean (SD)**	4.3 (2.0)	4.5 (2.0)	4.1 (2.0)	<0.001
**Diabetics, n (%)**	1687 (49.8)	781 (48.5)	906 (51.0)	0.15
**$0 co-pay, n (%)**	2695 (79.6)	1276 (79.3)	1419 (79.9)	0.64
**Days in study, mean (SD)**	571.6 (315.7)	546.2 (338.1)	594.6 (292.2)	<0.001
**No. of statin prescriptions, mean (SD)**	8.0 (5.2)	9.5 (5.9)	6.6 (4.1)	<0.001
**Days supply, n (%)**				
30 day	833 (24.6)	303 (18.8)	530 (29.8)	<0.001
60 day	2553 (75.4)	1307 (81.2)	1246 (70.2)	
Cholesterol (mg/dl)*, mean (SD)	186.8 (47.9) (n = 3292)	176.8 (42.2) (n = 1573)	195.9 (50.8) (n = 1719)	<0.001

Data are either n (%) per group with *P *Values determined from chi-squared tests or mean (standard deviation) with two sample *t *test *P *Values.

*Cholesterol values available on n = 3292 patients, multiply by 0.0259 to convert cholesterol to mmol/L.

Table 2 shows the adjusted risk ratios (RR) resulting from the log-binomial regression model. Patients with mostly 60-day statin fills were more likely to achieve at least 80% adherence than those who received mostly 30-day statin fills (RR 1.41, 95% confidence interval (CI) 1.28–1.55). Other factors significantly predicting adherence were increasing age, additional comorbidities, gender, and race. Insurance and co-payment were not significant predictors of adherence in this model. A paired comparison of the 1,412 patients who had received both 30- and 60-day fills demonstrated that when these individuals received 60-day fills, their adherence was 82.9% ± 52% compared to 73.9% ± 59% when receiving 30-day fills (*P *< 0.001).

**Table 2:** Multivariate Predictors of Statin Adherence

	Adjusted Risk Ratio* (95% Confidence Interval)
**60 versus 30 days supply**	1.40 (1.27–1.55)
**Age (per 10 year increase)**	1.07 (1.03–1.10)
**Female versus Male**	0.92 (0.86–0.98)
**Race (Black versus White)**	0.77(0.70–0.86)
**Race (Hispanic versus White)**	0.77(0.72–0.84)
**Race (Other versus White)**	0.98 (0.86–1.10)
**Each additional comorbidity**	1.04 (1.03–1.06)
**$0 Co-pay and uninsured**	1.06 (0.97–1.17)
**> $0 Co-pay and insured**	1.12 (0.99–1.27)
**> $0 Co-pay and uninsured**	0.91(0.79–1.05)

*Log-Binomial Regression used to calculate adjusted risk ratios

Patients in the 60-day supply group had a lower final serum cholesterol value of 185.3 ± 46.2 mg/dL (4.80 ± 1.20 mmol/L) compared to 191.5 ± 52.6 mg/dL (4.96 ± 1.36 mmol/L) for the 30-day supply group (*P *= 0.003). Multiple linear regression showed that lower adherence to statin medications was associated with an increase in final serum cholesterol values of 17.23 ± 1.64 mg/dL (0.45 ± 0.04 mmol/L) over the value predicted for the intercept (*P *< 0.001) (Table 3). Other significant predictors of final cholesterol value included age, gender, and the combination of having a median co-payment of greater than $0 and being uninsured. After adjusting for statin adherence demographics and copay, the effect of days supply on final cholesterol value was no longer significant (*P *= 0.09).

**Table 3:** Multivariate Predictors of Final Cholesterol Value (mg/dL)

	**Parameter Estimate (standard error)**	***P *Value**
Intercept*	175.36 (2.78)	<0.001

**Age (per 10 year increase)**	-5.79 (0.77)	<0.001
**Female versus Male**	12.60 (1.65)	<0.001
**Race (Hispanic versus White)**	-1.46 (1.97)	0.46
**Race (Black versus White)**	-0.01 (2.44)	0.99
**Race (Other versus White)**	2.30 (3.38)	0.50
**Mostly 30 Days Supply**	3.20 (1.91)	0.09
**<80% adherent**	17.23 (1.64)	<0.001
**$0 Co-pay and uninsured**	-3.18 (2.14)	0.14
**> $0 Co-pay and insured**	3.01 (3.19)	0.35
**> $0 Co-pay and uninsured**	10.48 (3.09)	<0.001

* Intercept is predicted cholesterol for a 50 year old white insured male with no co-payment, receiving mostly 60 day supply of statin medications, and achieving or exceeding 80% adherence. R^2 ^= 0.08

The average (s.d.) median co-payment for the 691 (20.4%) of the population with a non-zero median co-payment was $15.69 ($19.74). The other 2695 (79.6%) of the population filled at least half of their prescriptions at no cost through our robust indigent care prescription plan. In the population that was uninsured but did not qualify for free drug (n = 346) cholesterol levels were higher by 10 mg/dL than those who were uninsured but did qualify for free drug.

## Discussion

Our results demonstrate that patients receiving a larger quantity of statin medication at each fill achieved greater adherence, and that higher adherence was a significant predictor of lower serum cholesterol. In addition, patients who changed from a 30-day fill to a 60-day fill pattern had higher rates of adherence with the larger supply. Given the relationship of greater adherence with lower cholesterol levels, this suggests that an institutional pharmacy strategy of increasing the days supply of statins dispensed has the potential to favorably impact modifiable cardiac risk factors among vulnerable patients with limited resources, by facilitating adherence behavior. Since the major reason for nonadherence cited by patients is forgetfulness [17] the provision of a 60-day fill may facilitate the ability of patients to follow a regular medical regimen. These findings have important implications for other closed pharmacy systems and countries with universal prescription drug coverage, where the costs associated with the loss of co-payment may be offset by greater adherence and drug efficacy as well as institutional efficiency.

There are several strengths to our study. First, we assessed all patients within Denver Health who received multiple statin prescriptions and received follow-up cholesterol measurement. This assures that our results are broadly applicable within that patient population. Second, patients in our cohort were unaware that adherence would be assessed, and thus adherence behavior was not altered by adherence measurement, a common flaw of studies based on self-report, electronic monitoring, or pill counts. Most importantly, identifying systematic means of improving adherence among vulnerable populations is unique. Numerous studies have shown disparities in guideline-based therapies among minorities and the poor [18-21]. The provision of greater prescription quantity therefore represents a potential method for improving care in this population.

A significant fraction of patients with hyperlipidemia discontinue therapy within the first year [14] and never reinitiate therapy. Therefore our inclusion of patients during the time that they were actually filling their statin would indicate that we cannot generalize adherence rates from this study to the entire population of individuals who were intended to receive treatment. We excluded 33.3% or 1687 patients from analysis who only filled one statin prescription during the study period. This is not unexpected based on prior work that has shown that even in patients with known coronary artery disease up to 60% discontinue lipid-lowering medications in the first year [22].

Many similar studies have found a direct relationship between co-payment size and adherence [23,24]. We did not observe this relationship in our study. However, as noted above, the majority of our patients had a median co-payment of $0.

This study has a number of limitations. The use of medication acquisition to determine adherence likely overestimates actual adherence because it assumes that patients consume all of the medication for prescriptions that are filled. We did not analyze low-density lipoprotein (LDL) cholesterol levels because these data were not available for many patients. Moreover, the National Cholesterol Education Program (NCEP) guidelines for LDL-cholesterol targets have changed [25]. Therefore, assessment of total cholesterol provided the broadest applicability to our entire cohort. Since our study included both patients newly initiating treatment as well as those receiving ongoing treatment, we chose to use the cholesterol measurement at the end each patient's study period in order to best capture a value that reflected the adherence period observed. While other measurements for cholesterol, such as averaging all measurements over the study period, may have better captured the effect of adherence on cholesterol levels for those with ongoing therapy, it would not have been appropriate for those newly initiating therapy.

Finally, cholesterol level itself is a surrogate marker. We did not attempt to obtain cardiovascular (CV) outcomes for these patients, though recent data suggest that statin adherence is associated with a significant reduction in CV events and revascularization [17] and is therefore a valid surrogate outcome marker.

## Conclusion

This study demonstrates that providing patients with a larger statin fill size is associated with greater adherence as well as an increased likelihood of achieving lower cholesterol levels. Though numerous factors contribute to both statin adherence and cholesterol level, identifying an easily modifiable factor related to both may be useful to clinicians, pharmacies, and health care delivery systems. Pharmacy and institutional policies that purposely limit medication fill size to minimize medication wastage and maximize co-payment collection may be shortsighted. Further exploration of the policy and system changes necessary to make dispensing larger prescription amounts cost effective seems warranted.

## Competing interests

The author(s) declare that they have no competing interests.

## Authors' contributions

HAB conceived of the study, applied for funding, coordinated data acquisition, and drafted the manuscript. MJK helped draft the manuscript and formulate the discussion. PSM participated in the design of the study and reviewed the results and discussion. RAD performed the statistical analysis. JFS contributed the prescription adherence calculations and interpretation of the data. All authors contributed to, read, and approved the final manuscript.

## Pre-publication history

The pre-publication history for this paper can be accessed here:


